# Epidemiology, Etiology and Intervention Strategies for Peri-Partum Depression in Mothers

**DOI:** 10.3390/jcm12185822

**Published:** 2023-09-07

**Authors:** Silvia Cimino

**Affiliations:** Department of Dynamic, Clinical and Health Psychology, Sapienza, University of Rome, Via degli Apuli 1, 00186 Rome, Italy; silvia.cimino@uniroma1.it; Tel.: +39-064-9912

The prevalence of peri-partum depression (PPD) varies widely across countries, with rates ranging from 10% to 15% depending on the screening method used and the country studied [1]. Risk factors for PPD include depression and anxiety existing prior to pregnancy, previous psychiatric illness, poor marital relationships, stressful life events, negative attitude towards pregnancy, and lack of social support. PPD is associated with negative health-related behaviors and adverse outcomes for infants, children, and adolescents [2]. In low-income and middle-income countries, PPD is under-recognized and undertreated.

Experiencing depression, anxiety, or stress during pregnancy can impact the development of the infant by changing the conditions in the womb and how the fetus matures. Animal studies have offered a way to investigate the biological processes involved in this connection [3]. The disruption of the hypothalamic–pituitary–adrenal axis, which reflects stress reactivity, has been considered the most likely biological explanation for the harmful effects of maternal symptoms on infant development during pregnancy. Consistently high levels of glucocorticoid during pregnancy have been found to have negative effects in both animal models, where it leads to anxiety-like behaviors, and in laboratory models, where it is linked to reduced neural progenitor cell growth and the expression of genes associated with a higher susceptibility to psychiatric disorders. Furthermore, neuroimaging studies conducted on infants born to mothers with prenatal mental health issues have revealed differences in the structure and connectivity of brain regions specifically involved in evaluating threats and cognitive assessment [4,5]. These alterations in brain structures may potentially serve as an adaptive mechanism in the postpartum period, helping newborns better monitor a less responsive interactive partner.

Many strategies have been developed for the purpose of preventing and treating peri-partum depression in women. The academic community has not reached a unanimous agreement about the effectiveness of medications for mothers with depression during pregnancy, after delivery, and during breastfeeding. Therefore, non-pharmacological approaches have been tested, with positive results. For instance, activities like exercise, acupuncture, massage, and dietary supplements have been employed, as well as nutritional supplements, such as omega-3 and folic acid. In fact, omega-3 supplementation was effective at alleviating depressive-like symptoms in an animal model simulating pregnancy-related hormonal changes in PPD. On the other hand, psychological therapies have been developed. The use of cognitive–behavioral therapy (CBT) is one specific example [6,7,8]. CBT helps mothers establish healthier coping mechanisms and enhances their general wellbeing by identifying and changing harmful thinking patterns and behaviors that lead to depressive symptoms. A different example is the creation of support groups for women who are going through peri-partum depression [9,10,11]. In these groups, they may discuss their experiences, receive sympathy and validation, access helpful resources, and receive expert advice in a judgment-free setting. It is crucial to recognize that while therapy and support groups may be helpful for some mothers suffering from peri-partum depression, they may not be useful for everyone and could ignore underlying biological issues causing the depressive symptoms [12,13,14]. In these situations, a thorough approach to treatment that includes counseling and medicine may be required. Furthermore, it is essential for healthcare professionals to carry out thorough assessments to identify the underlying reasons of depression and make sure that the right therapies are put into place. It is possible for mothers to have a higher chance of overcoming this difficult illness and reaching good mental health by treating both the psychological and biological/genetic elements of peri-partum depression [13,14,15].

Another highly efficient method of preventing and treating mothers’ depression is home visiting (HV), which was developed and run by a team of gifted professionals under Selma Fraiberg’s guidance (for a seminal contribution from Italy, see [16]) [17,18]. In order to provide psychological treatment and preventative intervention for parents, newborns, and their developing parent–child interactions, Fraiberg worked with social workers, psychologists, nurses, and psychiatrists. Fraiberg and her colleagues emphasized the significance of dyadic caring interactions in the early stages of infant mental health. In addition to assisting parents in finding their missing child and avoiding or treating depressive symptoms, home visiting emphasizes a combination of social, emotional, and cognitive elements [19,20,21,22,23] and the quality of those interactions directly in the family’s home; forging an alliance or working relationship; meeting material needs; providing emotional support; guiding developmental progress; and acquiring life-coping skills and social support [24,25,26,27,28,29,30].

The bond between parents and infants has an important influence in the development of the neurobiology and the brain’s architecture, according to a more recent study. A person’s emotional responsiveness and stress-coping mechanisms later in life are significantly altered, according to Schore’s observation that “traumatic dysregulating levels of relational stress during the early stages of life exert an enduring detrimental epigenetic impact on the developing right brain” [31]. Parents (like depressed mothers) who lack implicit procedural memories [32] of being cared for in times of alarm or stress themselves risk becoming overwhelmed in the face of their infants’ distress, which can result in disruptive responses to the baby and thus continue the intergenerational cycle of misattuned, insensitive caregiving. Additionally, harsh and frightening parenting, often rooted in the caregiver’s own early history of unresolved fright, can contribute to the baby’s ongoing experience of the parent being a source of alarm [33,34,35,36].
